# The potential of ^1^H-MRS in CNS drug development

**DOI:** 10.1007/s00213-019-05344-7

**Published:** 2019-09-05

**Authors:** Alice Egerton

**Affiliations:** Department of Psychosis Studies, Institute of Psychiatry, Psychology & Neuroscience, King’s College London, De Crespigny Park, London, UK

**Keywords:** Glutamate, GABA, Magnetic resonance spectroscopy, Psychiatry, Drug development, Biomarkers

## Abstract

**Rationale:**

Proton magnetic resonance spectroscopy (^1^H-MRS) is a cross-species neuroimaging technique that can measure concentrations of several brain metabolites, including glutamate and GABA. This non-invasive method has promise in developing centrally acting drugs, as it can be performed repeatedly within-subjects and be used to translate findings from the preclinical to clinical laboratory using the same imaging biomarker.

**Objectives:**

This review focuses on the utility of single-voxel ^1^H-MRS in developing novel glutamatergic or GABAergic drugs for the treatment of psychiatric disorders and includes research performed in rodent models, healthy volunteers and patient cohorts.

**Results:**

Overall, these studies indicate that ^1^H-MRS is able to detect the predicted pharmacological effects of glutamatergic or GABAergic drugs on voxel glutamate or GABA concentrations, although there is a shortage of studies examining dose-related effects. Clinical studies have applied ^1^H-MRS to better understand drug therapeutic mechanisms, including the glutamatergic effects of ketamine in depression and of acamprosate in alcohol dependence. There is an emerging interest in identifying patient subgroups with ‘high’ or ‘low’ brain regional ^1^H-MRS glutamate levels for more targeted drug development, which may require ancillary biomarkers to improve the accuracy of subgroup discrimination.

**Conclusions:**

Considerations for future research include the sensitivity of single-voxel ^1^H-MRS in detecting drug effects, inter-site measurement reliability and the interpretation of drug-induced changes in ^1^H-MRS metabolites relative to the known pharmacological molecular mechanisms. On-going technological development, in single-voxel ^1^H-MRS and in related complementary techniques, will further support applications within CNS drug discovery.

## Introduction

Proton magnetic resonance spectroscopy (^1^H-MRS) is a non-invasive in vivo technique that can be used to measure regional concentrations of brain metabolites, including glutamate and γ-amino butyric acid (GABA). A major advantage of ^1^H-MRS in central nervous system (CNS) drug discovery is that it provides a translational technique, whereby the ability of pharmacological compounds to modulate a MRS-detectable metabolite of interest in laboratory animals can then be tested in healthy humans or patient populations using the same imaging biomarker. In parallel, key findings from clinical research can be translated back to the preclinical laboratory, to develop animal disease models and test potential compounds for their ability to modulate brain metabolite abnormalities associated with CNS disorders.

This article reviews the potential of ^1^H-MRS for CNS drug development, with a focus on the development of novel glutamatergic and GABAergic compounds for the treatment of psychiatric disorders. The review describes the relevance of ^1^H-MRS-quantifiable metabolites to drug discovery, before discussing potential applications at different stages of drug development. In this emerging area, the review provides examples of applications to date and then considers the current limitations and future directions for research.

## ^1^H-MRS metabolites: relevance to psychiatric drug discovery

In vivo ^1^H-MRS is an MRI-based neuroimaging approach, which is most commonly used to measure metabolites in a predefined three-dimensional voxel, prescribed in a brain region of interest rather than across the whole brain. Depending on the magnetic field strength of the MRI scanner and acquisition sequence, it is now possible to detect over 18 metabolites in an in vivo ^1^H-MRS spectrum (Pfeuffer et al. 1999). Both human and rodent ^1^H-MRS can measure several metabolites involved in neurotransmission, oxidative stress or inflammation, which are key targets of interest for CNS drug discovery.

### ^1^H-MRS metabolites involved in excitatory and inhibitory neurotransmission

Within drug discovery for psychiatric disorders, the majority of ^1^H-MRS research has focussed on glutamate and GABA. At lower field strengths of around 1.5 Tesla (which corresponds to most clinical MRI scanners), glutamate and its metabolite glutamine have overlapping resonances and are usually reported in combination, termed Glx (Hancu 2009). At higher field strengths of 3 Tesla and above (corresponding to MRI scanners for clinical research), it becomes increasingly possible to resolve the glutamate and glutamine signals (Mullins et al. 2008; Snyder and Wilman 2010; Terpstra et al. 2016; Tkac et al. 2001) and their resolution may be further improved with specialised pulse sequences (Bustillo et al. 2016; Mekle et al. 2009; Wijtenburg and Knight-Scott 2011; Zhang and Shen 2016). GABA is less abundant than glutamate and has overlapping resonances with other metabolites, and detection of GABA can be facilitated through application of spectral editing, typically using Mescher-Garwood point resolved spectroscopy (MEGA-PRESS) (Puts and Edden 2012). Glutamate and glutamine may also be measurable in GABA-edited MEGA-PRESS spectra although there are challenges around their reliable quantification (Sanaei Nezhad et al. 2018).

An important consideration is that ^1^H-MRS quantifies the total amount of MR visible metabolite in the voxel, so glutamate levels will reflect neurotransmission but also other cellular metabolic processes. Glutamate released from synapses is rapidly converted to glutamine in astrocytes for recycling to glutamate and GABA, and this accounts for approximately 80% of glutamine synthesis (Kanamori et al. 2002; Rothman et al. 2011; Rothman et al. 1999; Sibson et al. 1997; Sibson et al. 2001). Some studies have drawn inferences about glutamate, glutamine or GABA concentrations though investigating changes in their relative ratios. Elevations in the glutamine to glutamate ratio have been interpreted as increased glutamate turnover (Brennan et al. 2010; Bustillo et al. 2010; Xu et al. 2005), and regional glutamate to GABA ratios have been discussed in the context of excitatory-inhibitory (E/I) balance (Ajram et al. 2017; Cohen Kadosh et al. 2015; Colic et al. 2018; Ferri et al. 2017; Foss-Feig et al. 2017; Gu et al. 2019). Nonetheless, the cellular mechanisms that may underpin an observed difference in the ^1^H-MRS glutamine/glutamate or GABA/glutamate signal ratio are complex, and when interpreting metabolite ratios it is important to consider whether the observed differences in ratios are primarily driven by the numerator or denominator.

**Fig. 1:**
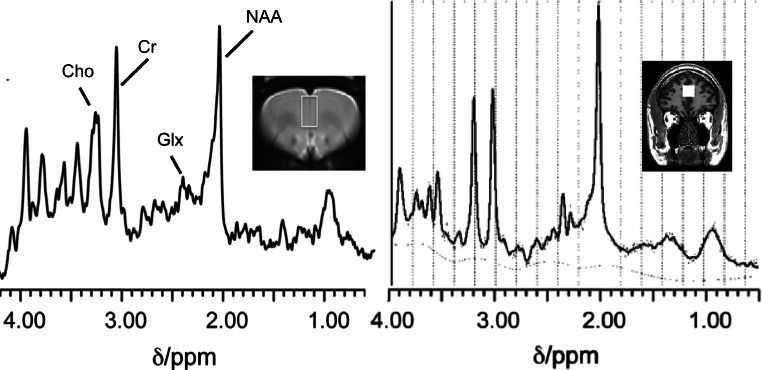
Representative ^1^H-MRS spectra from the rat (left) and human (right) medial prefrontal cortex. The images show the ^1^H-MRS voxel position in each species overlaid on the corresponding anatomical MRI image. In the rat, spectra were acquired under 1.0% isoflurane anaesthesia in a 3.8 × 2.2 × 2.0-mm voxel at 7 Tesla (Agilent Technologies Inc.) using a point-resolved spin-echo sequence (PRESS) with a repetition time (TR) of 3000 ms and an echo time (TE) of 24 ms.(Vernon et al. 2015) In man, spectra were acquired in a 20 × 20 × 20-mm voxel at 3 Tesla (GE MR750, General Electric Healthcare), using a PRESS sequence with TR = 2000 ms and TE = 35 ms. Representative spectra in each species, provided using LCModel software (Provencher 1993), show peaks for choline-containing compounds (Cho), creatine (Cr), glutamate and glutamine (Glx) and *N*-acetylaspartate (NAA)

At high field strengths, it may become possible to quantify additional glutamatergic metabolites in the ^1^H-MRS spectra, including glycine, serine and *n*-acetylaspartylglutamate (NAAG) (discussed in Harris et al. 2017) (Fig. 1). Due to their function as *N*-methyl-d-aspartate (NMDA) receptor co-agonists, glycine and serine are of interest to drug discovery in psychiatric disorders associated with NMDA receptor dysfunction, most prominently schizophrenia (Goff 2015; Moghaddam and Javitt 2012). Glycine and serine also act as inhibitory neurotransmitters through activating glycine receptors-chloride channels (Legendre 2001). In man, glycine has been measured at field strengths of 3 T or more using specialised sequences (Kaufman et al. 2009; Kim et al. 2017; Prescot et al. 2006; Tiwari et al. 2017), while the detection of serine remains more challenging (Harris et al. 2017).

NAAG, present in neurons and glia, is a neuromodulatory peptide that acts as an agonist at mGluR3 receptors to decrease neurotransmitter release (see Neale et al. 2011). NAAG can also act as a NMDA receptor antagonist (Bergeron et al. 2007) or agonist (Westbrook et al. 1986) depending on the cellular environment and subunit composition of the NMDA receptors, among other factors (Khacho et al. 2015). Compounds that increase NAAG, such as NAAG peptidase inhibitors, may have therapeutic effects that may be associated with mGluR2/3 agonism, including antipsychotic effects (Olszewski et al. 2004) or decreasing additive behaviours (Xi et al. 2010a, b). While it may be possible to isolate NAAG from the larger *n*-acetylaspartate (NAA) signal using spectral editing at human field strengths (Edden et al. 2007; Harris et al. 2017), few studies have used these techniques (Jessen et al. 2011; Landim et al. 2016; Rowland et al. 2013).

### ^1^H-MRS metabolites as markers of brain inflammation or oxidative stress

Also relevant to drug discovery in psychiatric disorders are ^1^H-MRS metabolites that act as antioxidants and markers of brain inflammation. Myo-inositol (mIns), a precursor of the phosphatidylinositol membrane lipids (Berridge and Irvine 1989) and choline-containing compounds (phosphocholine and glycerophosphocholine) are predominantly expressed in glial cells (Brand et al. 1993; Urenjak et al. 1993). Both mIns and choline have been interpreted as markers of glial activation, which may provide a proxy measure of inflammation (Bagory et al. 2012; Chang et al. 2004; Chang et al. 2013; Harris et al. 2015; Kirov et al. 2009). These metabolites could therefore be used as a marker for target engagement for anti-inflammatory drugs, for example in patients with traumatic brain injury (Harris et al. 2015), but also in psychiatric disorders associated with inflammation including depression (Caetano et al. 2005; Venkatraman et al. 2009) and schizophrenia (Plitman et al. 2016; Rowland et al. 2017). Potentially, mIns and choline could also be used as a biomarker to stratify such patients for clinical trials of anti-inflammatory treatments. Glutathione (GSH), an endogenous antioxidant, is also detectable in the ^1^H-MRS spectra and could provide a translational biomarker for antioxidant drug development (Conus et al. 2018).

^1^H-MRS can detect other several metabolites involved in brain regulatory processes such as myelination, membrane lipid metabolism and energy metabolism (reviewed in Duarte et al. 2012). These metabolites, including glucose, lactate, creatine and *N*-acetylaspartate (NAA), are of less interest to pharmacoMRS studies, but may be relevant for the creation or validation of animal models mimicking pathological changes observed in clinical populations. For example, the most abundant metabolite in the ^1^H-MRS spectra, NAA, is expressed almost completely in neurons. NAA is commonly interpreted as a marker of neuronal integrity, although the physiological roles of NAA in neuronal function remain largely unresolved (Ariyannur et al. 2013; Moffett et al. 2007). Psychiatric disorders including schizophrenia and bipolar disorder show regional NAA reductions (Brugger et al. 2011; Kraguljac et al. 2012; Schwerk et al. 2014) which could be recapitulated in animal models, although the extent to which NAA reductions may be reversed with pharmacological treatment is not clear.

## Potential applications of ^1^H-MRS in drug development

^1^H-MRS could potentially be applied to accelerate CNS drug discovery at various stages of the drug development pipeline, either alone or in combination with other imaging modalities (Wong et al. 2009). This is an emerging application of ^1^H-MRS within psychiatry. As such, the following section discusses the potential of this approach using examples where they are already available and considers the future developments that are required to support wider implementation. Box 1 provides selected examples of current ^1^H-MRS applications, and Box 2 summarises key considerations for future research.

Box 1: Selected examples of current applications of ^1^H-MRS in psychiatric research and CNS drug development

**Table:** 

Translation of rodent models to man *Example:* Increases in frontal or hippocampal glutamatergic metabolites occurring on ketamine administration, as a cross-species model of NMDA hypofunction in schizophrenia (Bojesen et al. 2018; Javitt et al. 2018; Kraguljac et al. 2017; Rowland et al. 2005; Stone et al. 2012).	
Understanding drug therapeutic mechanisms *Example:* Ketamine-induced changes in frontal or occipital glutamate in relation to antidepressant efficacy (Evans et al. 2018; Milak et al. 2016; Valentine et al. 2011).	
Target engagement *Example:* Increases in prefrontal glutamate metabolites and GABA on d-cycloserine administration (Kantrowitz et al. 2016).	
Preclinical model development *Example:* Effects of maternal immune activation, a risk factor for psychiatric disorders in offspring, on brain metabolites during rat developmental maturation (Vernon et al. 2015).	
Refining the therapeutic rationale *Example:* Linking pre-treatment glutamate levels to the degree of subsequent clinical response in first episode psychosis (Egerton et al. 2018; Szulc et al. 2013) or in bipolar disorder (Strawn et al. 2012).	

Box 2: Key considerations for ^1^H-MRS in psychiatric research and CNS drug development

**Table:** 

The extent to which ^1^H-MRS can detect dose-dependent drug effects at clinically relevant doses in man.	
Whether the signal change in pharmacoMRS studies is of sufficient magnitude and reliability to investigate the ability of second compound to attenuate the drug effect.	
Unclear relationships between pharmacological molecular mechanisms and ^1^H-MRS metabolite signal change.	
Issues around standardisation of data acquisition, read-out, intra and inter-site reliability, particularly for multicentre studies.	

.

### Biomarkers for target engagement

A first potential application of ^1^H-MRS is to employ pharmacologically induced changes in the ^1^H-MRS metabolite of interest (pharmacological MRS ‘pharmacoMRS’) as a biomarker of target engagement (TE). In preclinical studies, the most promising drug candidates could be selected from a series of compounds by examining their ability to modulate the target metabolite. A similar approach could assist dose selection of the candidate compound. Lead compounds could then be translated to human ^1^H-MRS studies using the same ^1^H-MRS TE biomarker for confirmation, before proceeding to clinical trials.

Application of ^1^H-MRS for TE requires prior confirmation that ^1^H-MRS has sensitivity to detect pharmacologically evoked changes in brain metabolites at relevant doses, and that these changes are consistent with established pharmacological drug effects (see Waschkies et al. 2014). Within psychiatric drug development, preclinical and clinical ^1^H-MRS studies have mainly investigated the effects of glutamatergic and GABAergic compounds (Table 1). Studies examining glutamatergic metabolites have variously reported changes in Glx, glutamate or glutamine. As discussed above, the reported glutamatergic metabolites will reflect the methodological features of the study that determine the ability to resolve signals from glutamine from glutamate, as well as potentially differential biological effects of the pharmacological challenge on glutamate versus glutamine concentrations. While there are some negative findings, overall, these studies indicate that ^1^H-MRS has sensitivity to detect the hypothesised drug effects, and that comparable effects can be observed in rodent and human pharmacoMRS.

**Table 1:** Preclinical and clinical in vivo pharmacoMRS studies of glutamatergic and GABAergic compounds

Author	Subject	Compound, design	Dosing	Voxel location	Results
*NMDA receptor antagonists*					
Iltis et al. (2009)	Rat	Phencyclidine vs. saline	10 mg/kg i.p.	PFC	Increase in Gln/Glu; NS for Glu, Gln
Lee et al. (2010)	Dog	Ketamine vs. pentobarbital anaesthesia	15 mg/kg i.v.	Striatum	Increase in Glx
Kim et al. (2011)	Rat	Ketamine vs. saline	30 mg/kg for 6 days	PFC	Increase in Glu
Napolitano et al. (2014)	Rat	Ketamine vs. saline	25 mg/kg i.p.	ACC/mFC	Increase in Gln in group-housed, decrease in GABA in isolated
Yoo et al. (2017)	Rat	MK-801 vs. saline	0.5 mg/kg for 6 days	PFC	NS for Glu, Gln
Sekar et al. (2018)	Rat	Memantine vs. vehicle	20 mg/kg/day i.p for 5 days	Hippocampus	NS for Glu, Gln, GABA
Servaes et al. (2019)	Rat	MK-801 or ebselen vs. saline	MK-801 0.3 mg/kg i.p., ebselen 10 mg/kg p.o. for 7 days	Striatum	NS for Glu, decrease in Gln in ebselen group.
Rowland et al. (2005)	Human (HV)	Ketamine vs. placebo, crossover	Loading 0.27 mg/kg over 10 min; maintenance 0.00225 mg/kg/min for up to 2 h	ACC	Increase in Gln during loading dose, NS for Glu
Valentine et al. (2011)	Human (MDD)	Ketamine vs. saline pre-post	0.5 mg/kg over 40 min	OCC	NS for Glu, Gln, GABA
Taylor et al. (2012)	Human (HV)	Ketamine vs. placebo parallel group	0.5 mg/kg over 40 min	ACC	NS for Glu or Glx
Stone et al. (2012)	Human (HV)	Ketamine, pre-post	0.26 mg/kg bolus then 0.42 mg/kg/h	ACC (Glu) and thalamus (GABA)	Increase in ACC Glu, NS for Glx or GABA, 25–35 min after bolus
Milak et al. (2016)	Human (MDD)	Ketamine pre-post	0.5 mg/kg over 40 min	mPFC	Increase in Glu and GABA over 40 min
Rodriguez et al. (2015)	Human (OCD)	Ketamine vs. placebo, crossover	0.5 mg/kg over 40 min	mPFC	Increase in GABA; NS for Glx over 60 min
Li et al. (2017)	Human (HV)	Ketamine vs. placebo parallel group	0.5 mg/kg over 40 min	pgACC and aMCC	Increase in Gln/Glu in pgACC at 24 h but not 1 h post-ketamine
Kraguljac et al. (2017)	Human (HV)	Ketamine pre-post	0.27 mg/kg over 10 min, then 0.25 mg/kg/h for 50 min	Left hippocampus	Increase in Glx
Bojesen et al. (2018)	Human (HV)	S-Ketamine, pre-post	Loading 0.25 mg/kg for 20 min, maintenance 0.125 mg/kg for 20 min	ACC, thalamus	NS for Glu, Glx or Gln
Javitt et al. (2018)	Human (HV)	Ketamine vs. placebo, parallel group.	0.23 mg/kg for 1 min, then 0.58 mg/kg/h over 30 min, then 0.29 mg/kg/h over 29 min.	ACC (mPFC)	Increase Glx over first 15 min, NS between 15 and 60 min
Evans et al. (2018)	Human (HV and MDD)	Ketamine vs placebo, crossover	0.5 mg/kg over 40 min	pgACC	Glu NS in both HV and MDD at 24 h post-ketamine
*NMDA glycine site agonists (direct or indirect)*					
Kaufman et al. (2009)	Human (HV)	Glycine	0.2 to 0.8 g/day for 2 weeks	OCC	Increase in Gly; NS for Glu
Strzelecki et al. (2015b)	Human (SCZ)	Sarcosine vs. placebo parallel group	2 g/day for 6 months	Left frontal white matter	Increase in Glx
Strzelecki et al. (2015c)	Human (SCZ)	Sarcosine vs. placebo parallel group	2 g/day for 6 months	Left dlPFC	Glx NS
Strzelecki et al. (2015a)	Human (SCZ)	Sarcosine vs. placebo parallel group	2 g/day for 6 months	Left Hippocampus	Decrease in Glx
Kantrowitz et al. (2016)	Human	d-Cycloserine, pre-post	1000 mg	mPFC	Increase in Glx
*N-acetylcysteine*					
Durieux et al. (2015)	Mouse	*N*-acetylcysteine vs. vehicle	150 mg/kg i.p.	Left striatum	Decrease Glu
das Neves Duarte et al. (2012)	Mouse	*N*-acetylcysteine	2.4 g/L in drinking water during development	Anterior cortex	Decrease Gln and Gln:Glu; Glu NS
Schmaal et al. (2012)	Human (cocaine-dependent)	*N*-acetylcysteine vs. placebo crossover	2.4 g single oral dose	ACC	Decrease Glu
Das et al. (2013)	Human (MDD)	*N*-acetylcysteine vs. placebo, parallel group	2 g/day for 12 weeks	mPFC	Increase Glx
Conus et al. (2018)	Human (EP)	*N*-acetylcysteine vs. placebo, parallel group	2.7 g/day for 6 months	mPFC	Glu, Gln, Gln:Glu NS
Schulte et al. (2017)	Human (smokers)	*N*-acetylcysteine vs. placebo, parallel group	2.4 g/day for 14 days	ACC	Glx, GABA NS
McQueen et al. (2018)	Human (SCZ)	*N*-acetylcysteine vs. placebo crossover	2.4 g single oral dose	ACC, right caudate	Decrease Glx in ACC, Glu NS.
Girgis et al. (2019)	Human (HV and SCZ)	*N*-acetylcysteine, pre-post	2.4 g single oral dose	dACC, mPFC	Glu, Glx, NS
O'Gorman Tuura et al. (2019)	Human (HV)	*N*-acetylcysteine vs no intervention, crossover	5 g i.v. over 1 h	PFC, striatum	Striatum: decrease Glx, Gln; PFC decrease Glx; Glu NS.
*Riluzole*					
Waschkies et al. (2014)	Rat	Riluzole vs. vehicle	3, 6, and 12 mg/kg i.p	PFC, striatum	PFC and striatum decrease Glu
Rizzo et al. (2017)	Rat	Riluzole vs. vehicle	6 mg/kg/day i.p, 15 days	Left mPFC, left striatum	In hypertensive but not control rats, decrease PFC Glu and Gln; GABA NS
Brennan et al. (2010)	Human (BPD)	Riluzole, pre-post	100-200 mg/day for 6 weeks	ACC, POC	Increase in Gln/Glu between days 0–2
Ajram et al. (2017)	Human (HV and ASD)	Riluzole vs placebo, crossover	50 mg oral single dose	dlPFC	Increased GABA/GABA + Glx in HV; Decreased GABA/GABA + Glx in ASD
Pillinger et al. (2019)	Human (HV and SCZ)	Riluzole, pre-post	50 mg twice daily for 2 days	ACC	Group by condition interaction related to Glx decrease in SCZ and increase in HV
*Other glutamatergic drugs*					
Waschkies et al. (2014)	Rat	MSO vs. vehicle	50, 100, and 200 mg/kg i.p.	PFC, striatum	PFC: dose-dependent decrease Glu increase Gln; striatum: decrease GABA increase Gln
Godlewska et al. (2018)	Human (BPD)	Lamotrigine, pre-post	TAU, 10–12 weeks	ACC	Glx NS
Umhau et al. (2010)	Human (alcohol dependence)	Acamprosate, pre-post	oral loading followed by 1998 mg daily for 4 weeks	ACC	Decrease Glu
Frye et al. (2016)	Human (alcohol dependence)	Acamprosate, pre-post	4 weeks	ACC	Decrease Glu
*GABAergic drugs*					
Waschkies et al. (2014)	Rat	Vigabatrin vs. vehicle	30, 100, and 300 mg/kg i.p.	PFC, striatum	Dose-dependent increase in GABA in PFC and striatum, decrease in Glu in PFC, increase in Gln in striatum
De Graaf (2006)	Rat	Vigabatrin pre-post	750 mg/kg, i.v.	Cortex	Increase GABA
Patel et al. (2006)	Rat	Vigabatrin vs. no treatment	0.5 g/kg, i.p., 24 h before study	Cortex	Increase GABA
Waschkies et al. (2014)	Rat	3-MP vs. vehicle	20, 30, and 40 mg/kg i.p.	PFC, striatum	Dose-dependent decrease GABA in striatum; PFC GABA NS; decrease PFC Glu; Gln NS
Waschkies et al. (2014)	Rat	Tiagabine vs. vehicle	10, 20, and 40 mg/kg per os	PFC, striatum	Increase GABA and Gln in striatum; decrease Glu PFC
Myers et al. (2014)	Human (HV)	Tiagabine pre-post	15 mg single oral dose	OCC, limbic region	NS GABA

*ACC* anterior cingulate cortex, *ASD* autism spectrum disorder, *aMCC* anterior midcingulate cortex, *BPD* bipolar disorder, *dlPFC* dorsolateral prefrontal cortex, *EP* early psychosis, *GABA* γ-amino-butyric acid, *Glu* glutamate, *Gln* glutamine, *Gly* glycine, *Glx* glutamate plus glutamine, *HV* healthy volunteers, *i.p.* intraperitoneal, *i.v.* intravenous, *MDD* major depressive disorder, *3-MP* 3-mercaptopropionate, *mPFC* medial prefrontal cortex, *MSO* methionine sulfoximine, *NS* non-significant, *OCD* obsessive compulsive disorder, *OCC* occipital cortex, *PFC* prefrontal cortex, *pgACC* pregenual anterior cingulate cortex, *POC* parietal-occipital cortex, *SCZ* schizophrenia, *TAU* treatment as usual

A number of ^1^H-MRS studies have now examined the effects of administration of NMDA antagonists such as ketamine (Table 1), which are expected to increase extracellular glutamate (Moghaddam et al. 1997). Ketamine has mainly been investigated as an experimental model of NMDA hypofunction in schizophrenia (Bojesen et al. 2018; Javitt et al. 2018; Kraguljac et al. 2017; Rowland et al. 2005; Stone et al. 2012), or in relation to its antidepressant effects (Evans et al. 2018; Li et al. 2017; Milak et al. 2016; Taylor et al. 2012; Valentine et al. 2011). As hypothesised, several studies have observed increases in the glutamate, glutamine or Glx signal following NMDA antagonist administration, both in experimental animals (Iltis et al. 2009; Kim et al. 2011; Lee et al. 2010; Napolitano et al. 2014) and in man (Javitt et al. 2018; Kraguljac et al. 2017; Li et al. 2017; Milak et al. 2016; Rowland et al. 2005; Stone et al. 2012). However, some studies have not observed any changes in glutamatergic metabolites (Bojesen et al. 2018; Evans et al. 2018; Rodriguez et al. 2015; Sekar et al. 2018; Servaes et al. 2019; Taylor et al. 2012; Valentine et al. 2011; Yoo et al. 2017). Increases in Glx in the human medial prefrontal cortex were also observed after a single administration of NMDA receptor glycine site partial agonist d-cycloserine, which like ketamine is of interest as an antidepressant (Kantrowitz et al. 2016). Conversely, the few studies examining administration of glycine site agonists, which may be predicted to reduce glutamate levels via increased NMDA receptor activation, have produced mixed results; no difference in glutamate was apparent after 2 weeks of glycine administration (Kaufman et al. 2009), and in patients with schizophrenia, 6 months of sarcosine produced regionally dependent changes in Glx (Strzelecki et al. 2015a, b). Decreases in glutamatergic metabolites (glutamate, glutamine or Glx) have been observed across species following administration of several other compounds, including *n*-acetylcysteine (das Neves Duarte et al. 2012; Durieux et al. 2015; McQueen et al. 2018; O'Gorman Tuura et al. 2019; Schmaal et al. 2012), riluzole (Rizzo et al. 2017; Waschkies et al. 2014) and acamprosate (Frye et al. 2016; Umhau et al. 2010), although again not without some negative or discrepant findings (Table 1) (Brennan et al. 2010; Das et al. 2013; Girgis et al. 2019; Pillinger et al. 2019; Schulte et al. 2017).

There are fewer studies examining GABAergic compounds. In rats, ^1^H-MRS GABA levels show the predicted increases on administration of the GABA-transaminase inhibitor vigabatrin (de Graaf et al. 2006; Patel et al. 2006; Waschkies et al. 2014), or GABA transporter inhibitor tiagabine (Waschkies et al. 2014), and decreases on administration of 3-mercaptopropionate (3-MP), a glutamic acid decarboxylase inhibitor (Waschkies et al. 2014). The only human ^1^H-MRS study investigating administration of a GABAergic compound, tiagabine, did not observe significant change in GABA (Myers et al. 2014).

To investigate whether ^1^H-MRS glutamate and GABA are sensitive to dose-dependent effects, Waschkies et al. (2014) examined a range of doses of five glutamatergic or GABAergic compounds (vigabatrin, 3-mercaptopropionate, tiagabine, methionine sulfoximine and riluzole) in rats. Dose-related effects were detected in the frontal cortex and striatum, which should be confirmed in further rodent studies. To date, human ^1^H-MRS studies of acute glutamatergic or GABAergic drug effects have only investigated single doses (Table 1). Whether ^1^H-MRS has sensitivity to detect dose-related changes in glutamate and GABA metabolites in man within acceptable human dose ranges therefore remains to be established.

Research into the development of glutamate-targeting drugs for schizophrenia has provided some examples of how ^1^H-MRS TE biomarkers might be applied in early phase clinical trials. The variability with which symptoms of schizophrenia improve during adjunctive glycine treatment may partially depend on individual differences in glycine CNS penetration (Kaufman et al. 2009). In healthy volunteers, Kaufman et al. (2009) showed that ^1^H-MRS was able to detect increases in brain glycine levels over 2 weeks of glycine administration. In further clinical trials of compounds designed to increase glycine concentrations in patients, ^1^H-MRS could be used to measure the extent of TE and relationship with efficacy. As a similar example, ^1^H-MRS has been applied as a marker of TE in a clinical trial of the antioxidant and glutathione (GSH) precursor *n*-acetylcysteine (Conus et al. 2018). In patients with early psychosis, significant increases (22.6%) in medial prefrontal cortex GSH levels were observed after 6 months of administration of *N*-acetylcysteine but not placebo, indicating *N*-acetylcysteine TE (Conus et al. 2018). A final example concerns the attenuation of ketamine-induced increases in ^1^H-MRS Glx as a biomarker for TE in early stage clinical trials of novel glutamatergic compounds (Javitt et al. 2018). This multicentre study detected ketamine-induced increases in Glx in the medial frontal cortex with a moderate effect size of *d* = 0.6, although this may not provide sufficient power for reliably detecting subsequent reversal by glutamatergic compounds (Javitt et al. 2018). Similar applications of ^1^H-MRS may be strengthened by the combination of ^1^H-MRS with other biomarkers of TE in a multimodal approach as well as technical refinements to increase sensitivity.

### Preclinical model development

A second application of ^1^H-MRS within drug development is to translate findings in clinical populations back to animal models, to determine the validity of the model in recapitulating the neurochemical abnormality seen in the patient population using the same imaging technique. As a next step, the ability of pharmacological compounds to restore levels of ^1^H-MRS metabolites to control levels may then be used as a translational predictive biomarker for efficacy.

A key advantage of ^1^H-MRS in animal models is that data can be acquired repeatedly in the same animal, to provide longitudinal studies of drug effects. For example, this has allowed investigation of the effects of psychoactive bacteria on brain neurometabolites and how these respond after cessation of treatment (Janik et al. 2016), the changes in brain metabolites in rats exposed to maternal immune activation as they develop through adolescence into adulthood (Vernon et al. 2015) and the effects of pharmacological interventions on the emerging developmental abnormalities in genetically modified compared to wild-type mice (das Neves Duarte et al. 2012). These types of within-subject studies may also bring statistical advantages and reduce the numbers of experimental animals required.

### Refining the therapeutic rationale and patient stratification

Within psychiatric disorders, ^1^H-MRS research has not revealed a clear abnormality in brain metabolites that could be used diagnostically (as is the case for other biological measures). ^1^H-MRS glutamate studies comparing patients with schizophrenia to healthy volunteers have produced mixed findings, which may be related to illness stage, antipsychotic effects or other factors (Marsman et al. 2011; Merritt et al. 2016). In major depressive disorder (Moriguchi et al. 2018) and bipolar disorder (Taylor 2014), ^1^H-MRS studies may also indicate subgroups of patients within the diagnosis. Understanding this biological heterogeneity within a given diagnostic category may be a crucial factor in successful drug development. This raises the possibility that ^1^H-MRS measures could be used as a biomarker for intermediate phenotypes, to identify the subgroup of patients who are more likely to respond to a particular pharmacological intervention, and to stratify patients for clinical trials of this intervention.

While this is an emerging area of research, there is some early evidence to support the use of ^1^H-MRS in refining the therapeutic rationale and clinical trial stratification. Measures of glutamatergic ^1^H-MRS metabolites prior to starting a pharmacological treatment have been associated with the subsequent degree of response to a number of compounds, including antipsychotics in patients with first episode psychosis or established schizophrenia (Egerton et al. 2018; Szulc et al. 2013), ketamine in major depressive disorder (Salvadore et al. 2012) or valproate in bipolar disorder (Strawn et al. 2012). However, although these studies provide information on biological factors that may influence the degree of response to glutamate-acting drugs, significant further work is needed before ^1^H-MRS can be used to pre-select patient subgroups for stratified clinical trials. This would require reproducible data to define cut-off values for the level of the metabolite of interest that can most accurately predict the subgroup likely respond to the intervention and knowledge of the accuracy of the prediction. While ^1^H-MRS metabolite levels alone may not provide sufficient predictive accuracy for stratification, potentially the degree of accuracy could be improved through combination with other predictive variables (Egerton et al. 2018). Moreover, in the context of large clinical trials involving multiple recruitment sites, the non-trivial issues of standardisation of ^1^H-MRS acquisition and data values for patient selection across sites would be necessary. Research into the development of glutamatergic drugs for depression, schizophrenia or other disorders has revealed inverted U or non-linear dose-response relationships (Abdallah et al. 2018b; Foss-Feig et al. 2017). This suggests that measurement of pre-treatment levels of glutamatergic function may be important in identifying patient subgroups more likely to respond to novel glutamate-acting drugs, but may also complicate the use of ^1^H-MRS glutamate measures for patient stratification or dose selection.

Other studies have found no relationship between pre-treatment metabolite levels and response, but instead indicate that the degree of change in metabolite levels during treatment may mediate the extent of symptomatic improvement (Brennan et al. 2017; de la Fuente-Sandoval et al. 2013; Godlewska et al. 2018; Goff et al. 2002). In addition to providing mechanistic information, these studies are interesting in that they may suggest that early changes in glutamate metabolites occurring within the first 1–2 weeks of treatment may predict longer-term clinical outcomes. Such treatment-emergent biomarkers could be used in adaptive clinical trial design, where early ‘brain-level’ indicators of response could inform the treatment paradigm. As this approach would centre on the within-subjects change in ^1^H-MRS metabolite level rather than an individual value at a single time-point, some issues relating to standardisation of ^1^H-MRS data across multiple sites may be reduced.

## General limitations and future directions

A main limitation of ^1^H-MRS is that it measures the total voxel concentration of the MR-visible metabolite, across all cellular compartments. In glutamatergic or GABAergic drug development studies, this indirect measure of TE may limit the sensitivity and interpretation, as the relationship between interaction at the molecular drug target and the change in the total MR-visible glutamate or GABA signal may be non-linear or uncertain. As ^1^H-MRS is a technique that is translatable across species, combination with invasive methodologies in rodents such as microdialysis or fast-scan cyclic voltammetry could aid interpretation of the ^1^H-MRS signal. Where feasible, ^1^H-MRS could also be combined with more direct measures of target engagement in both humans and other animals, such as glutamate receptor occupancy as measured using positron emission tomography (PET) (Gruber and Ametamey 2017).

There is also on-going development of complementary MRS approaches that may provide deeper information on glutamatergic signalling. The far more technically challenging technique of carbon-13 magnetic resonance spectroscopy (^13^C-MRS) can measure glutamate-glutamine cycling by following the flow of a ^13^C isotope through the tricarboxylic acid cycle (see Rothman et al. 2011). To date, the application of ^13^C-MRS has been limited by technical complexity, sensitivity, spatial resolution and other factors, but with future technological advances, this technique may be of significant application to glutamatergic drug development. Indeed, a recent human study of ketamine using ^13^C-MRS found increases in prefrontal ^13^C glutamine enrichment, indicating increased glutamate-glutamine cycling (Abdallah et al. 2018a).

Also of interest is functional ^1^H-magnetic resonance spectroscopy (fMRS), which measures the dynamic change in the ^1^H-MRS metabolite signal occurring in response to a stimulus. Glutamate fMRS studies have detected stimulus-induced increases averaging 7% across experimental designs, and to the order of 13% in event-related studies (Mullins 2018). The greater magnitude of change detected in event-related compared to stimulus-block designs may relate to the relative rapidity of glutamate dynamics within the timeframe of the stimulation block, during which habituation, adaptation or homeostatic processes may occur (Apsvalka et al. 2015; Jelen et al. 2018). There are several potential explanations for magnitude of change in the fMRS signal, including increases in glutamate production from glucose oxidative metabolism (Mangia et al. 2007), compartmental shifts in glutamate from less (presynaptic vesicles) to more (extracellular) MRS-visible pools (Jelen et al. 2018; Kauppinen et al. 1994; Mullins 2018) and influences of blood oxygen level dependent (BOLD)-like effects on the signal amplitude (Apsvalka et al. 2015; Jelen et al. 2018). Pharmacological modulation of glutamate dynamics using fMRS is yet to be investigated and will be an interesting area for future research.

While the studies included in this review have investigated 1H-MRS metabolites serially in single voxels, it is also possible to measure multiple voxels simultaneously using MR spectroscopic imaging (MRSI). While initially limited to the more easily detectable metabolites such as NAA, ^1^H-MRSI sequences are now available to measure Glx (Ding et al. 2015; Gasparovic et al. 2011; Steel et al. 2018), glutamate and glutamine (Goryawala et al. 2016; Henning et al. 2009) and GABA (Moser et al. 2019) across large volumes of interest or the whole brain with a feasibly short acquisition time. Within psychiatric disorders, ^1^H-MRSI is therefore able to map the spatial distribution of metabolite abnormalities, which could provide richer regional information for future drug development. Finally, glutamate drug development could exploit the recent MRI technique of glutamate chemical exchange saturation transfer (GluCEST) (Cai et al. 2012), which has greater sensitivity, spatial and temporal for glutamate measurement than ^1^H-MRS.

^1^H-MRS studies in rodents are usually limited by the necessary use of anaesthesia to reduce stress to the animal and movement in the scanner, which will affect several aspects of brain physiology including metabolite concentrations (Makaryus et al. 2011) and could lead to divergence between results of preclinical and clinical studies. Under a typical protocol, in vivo ^1^H-MRS scanning takes approximately 1 hour per rat and can be associated with high costs of preclinical MRI equipment access or purchase, meaning it is probably impractical for initial drug screening. For a simple between-subjects group comparison, Waschkies et al. (2014) estimate that eight animals per group are required to detect a 6% change in glutamate, 12% change in GABA or 16% in glutamine (power = 80%; *α* = 5%), which is comparable to group sizes commonly required for invasive metabolite measurement. The authors also report good consistency of ^1^H-MRS measurement over time and across different batches of animals (Waschkies et al. 2014), which should be also established in other laboratories employing preclinical ^1^H-MRS. Despite the higher MR field strengths available for preclinical studies, the small voxel sizes required in relation to the rodent brain can limit detection of less abundant metabolites.

For human studies, participation in ^1^H-MRS requires that volunteers meet standard MRI inclusion criteria, such as an absence of implanted metallic objects. Typically, a ^1^H-MRS data acquisition to measure glutamate in a single voxel of ~ 8 ml would take about 10 minutes, with smaller voxels requiring longer acquisition times to obtain an equivalent signal to noise ratio. The short length of scan can generally be well tolerated by most individuals and patient groups. ^1^H-MRS data can be acquired as part of a set of MRI acquisition sequences, for example permitting MRS metabolite data to be investigated in relation to brain resting or functional activity during the same session. As mentioned above, it may be that multimodal imaging data could provide more reliable or accurate estimates for target engagement or patient stratification, as well as providing more comprehensive information on drug mechanism, and application of ^1^H-MRS to larger clinical studies across several study sites will benefit from protocol standardisation and evaluation of both inter- and intra-site reliability. Finally, with the availability of higher field strength MRI scanners for human research studies, it becomes increasingly possible to resolve lower concentration metabolites such as GABA or glutathione and separate glutamate and glutamine resonances under optimal acquisition sequences.

### Conclusion

There has been a growing interest in applying ^1^H-MRS to CNS drug discovery, including in the development of glutamate-acting compounds for psychiatric disorders. A key advantage of ^1^H-MRS is that it is a non-invasive technique that can directly translate findings from rodents to man using the same imaging biomarker which can be acquired repeatedly within subjects. An increasing number of experiments across species indicate that ^1^H-MRS has sensitivity to measure the predicted pharmacological effects of glutamatergic and GABAergic compounds at clinically relevant doses, and there are early indications that some ^1^H-MRS metabolite measures may contribute to identifying patient subgroups for stratified approaches to clinical trials. This research would be assisted through further methodological studies to measure and improve the reliability and sensitivity of ^1^H-MRS metabolite measurements, especially for multicentre application. On-going technological and methodological development, including high field strength MRI, optimised acquisition sequences, ^13^C-MRS and fMRS, may further support applications of MRS in CNS drug discovery.
